# Synthesis, crystal structure, and *in silico* mol­ecular docking studies of 4-hy­droxy-3,5-di­meth­oxy­benzaldehyde (6-chloro­pyridazin-3-yl)hydrazone monohydrate

**DOI:** 10.1107/S205698902500252X

**Published:** 2025-03-25

**Authors:** Muhammed Rafi Ummer, M. NizamMohideen, Mohammed Nazrudeen Noorulla, Abubacker Sidhik Moolan Khaja

**Affiliations:** a Post-Graduate and Research Department of Chemistry The New College University of Madras,Chennai 600 014 Tamilnadu India; b Department of Physics The New College Chennai 600 014 University of Madras,Tamil Nadu India; Universität Greifswald, Germany

**Keywords:** crystal structure, hydrazone, pyridazine, syringaldehyde, mol­ecular docking, EGFR kinase, hydrogen bonding

## Abstract

In the title compound, C_13_H_13_ClN_4_O_3_·H_2_O, the organic mol­ecule has an *E* configuration with regard to the C=N bond of the hydrazone bridge. The phenyl and pyridazine rings subtend a dihedral angle of 2.1 (1)° between their mean planes, while the hydrazone moiety makes dihedral angles of 1.6 (2) and 3.0 (2)°, respectively, with these aromatic rings. This renders the entire mol­ecule comparably flat. A C—H⋯N hydrogen bond generates an inversion dimer with a large *R*_2_^2^(14) ring motif. Within this ring, a further C—H⋯N hydrogen bond establishes a smaller *R*_2_^2^(8) ring. The mol­ecules of a dimer are thereby firmly linked by four hydrogen bonds. A bifurcated O—H⋯(O,O) hydrogen bond is formed between a water hydrogen atom and the hydroxyl and meth­oxy oxygen atoms of an adjacent mol­ecule, leading to the formation of an *R*_2_^1^(5) membered ring. C—H⋯π and face-to-face π–π stacking inter­actions are also present in the two-dimensional framework, which may be of relevance for the packing. In a complementary analysis, the compound was docked *in silico* to EGFR and HER2 receptors and the results imply that the compound targets EGFR preferentially over HER2.

## Abstract 2

The synthesis, mol­ecular X-ray structure and *in silico* EGFR and HER2 mol­ecular docking studies are reported for 4-hy­droxy-3,5-di­meth­oxy­benzaldehyde (6-chloro­pyridazin-3-yl)hydrazone hydrate, which constitutes a new pyridazine-based compound incorporating a syringaldehyde moiety by condensation of the hydrazine linker with the aldehyde function.

## 1. Chemical context

Hydrazone compounds are known to be associated with a wide spectrum of biological and medicinal applications, such as anti­microbial, anti­convulsant, analgesic and anti-inflammatory activities (NizamMohideen *et al.*, 2019 ▸). Compounds that target the epidermal growth factor receptor (EGFR) and the human epidermal growth factor receptor 2 (HER2) are known as tyrosine kinase inhibitors (TKIs) include gefitinib, erlotinib, neratinib, and afatinib, which act as anti­cancer agents (Uribe *et al.*, 2021 ▸; Weinberg *et al.*, 2020 ▸). However, these inhibitors show side effects and have adverse negative impacts on the patient’s health (Riecke & Witzel, 2020 ▸). As a result, new EGFR inhibitors need to be developed that would be more effective with less toxicity. Pyridazine derivatives have been the subject of extensive study in recent years, and the results have demonstrated a broad spectrum of pharmacological actions, including anti­depressant (Komkov *et al.*, 2015 ▸), COX-2 inhibitor (Harris *et al.*, 2004 ▸) and anti­cancer properties (Rafi *et al.*, 2019 ▸; Ahmad *et al.*, 2010 ▸). Commercially available physiologically active compounds with pyridazine as a structural component are hydralazine (vasodilator), minaprine (anti­depressant), and azelastine (bronchodilator) (Contreras *et al.*, 1999 ▸; del Olmo *et al.*, 2006 ▸). Our current research is focused on the synthesis of a new compound containing a pyridazine unit and a syringaldehyde derived moiety linked by a hydrazone motif, which may potentially act as a better TKI.
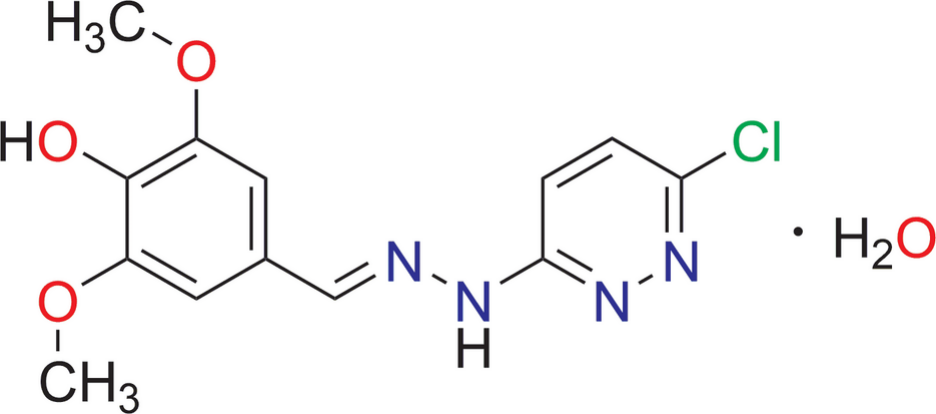


## 2. Structural commentary

**Figure 1:**
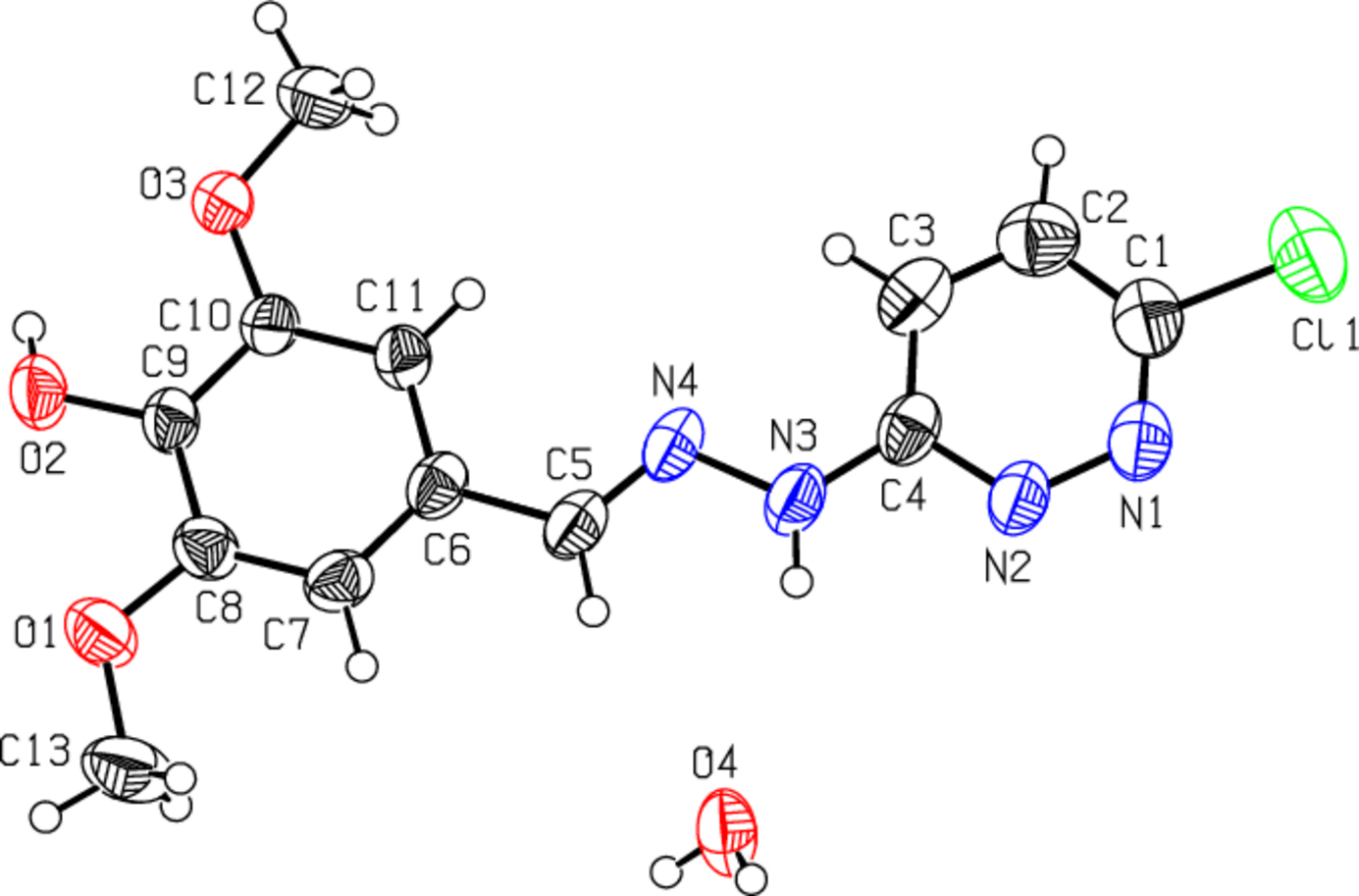
The mol­ecular structure of the title compound, with atom labelling. Displacement ellipsoids are drawn at the 50% probability level.

The mol­ecular structure of the title compound is illustrated in Fig. 1 ▸. The organic mol­ecule features a central, essentially planar region flanked on either side by a chlorine-substituted pyridazine, and a tris­ubstituted phenyl ring and it crystallized together with a water mol­ecule. The phenyl and pyridazine rings (C6–C11 and N1/N2/C1–C4, respectively) are each planar with a dihedral angle of 2.1 (1)° between their mean planes. The mean plane through the hydrazone unit (N3/N4/C5) forms angles of 1.6 (2) and 3.0 (2)° with the phenyl and pyridazine rings, respectively. In the title compound, the hydrazone mol­ecule adopts an *E* configuration with regard to the hydrazone bridge N4=C5, with torsion angles N3—N4—C5—C6 of −178.3 (2)° and C4—N3—N4—C5 of 178.5 (2)°, which are consistent with the *trans* relationship in the central moiety (Table 1 ▸). The bond lengths and angles in the hydrazone functional group of the title compound are comparable with the values reported for related structures (NizamMohideen *et al.*, 2019 ▸; Prabhu *et al.*, 2011 ▸). The chlorine and hy­droxy oxygen atoms deviate by −0.05 and 0.15 Å, respectively, from their pyridazine and phenyl ring mean planes. The C4—N3 and C5=N4 bond lengths differ by 0.075 Å, whereby these two bonds may be assigned as localized single and double bonds, respectively, while some resonance effects cannot be firmly excluded. One of the two meth­oxy groups is more coplanar with the C5–C11 phenyl ring than the other one, which deviates somewhat from the benzene ring plane, with torsion angles C12—O3—C10—C11 of 5.9 (3) Å and C13—O1—C8—C7 of 8.3 (3) Å.

## 3. Supra­molecular features

**Figure 2:**
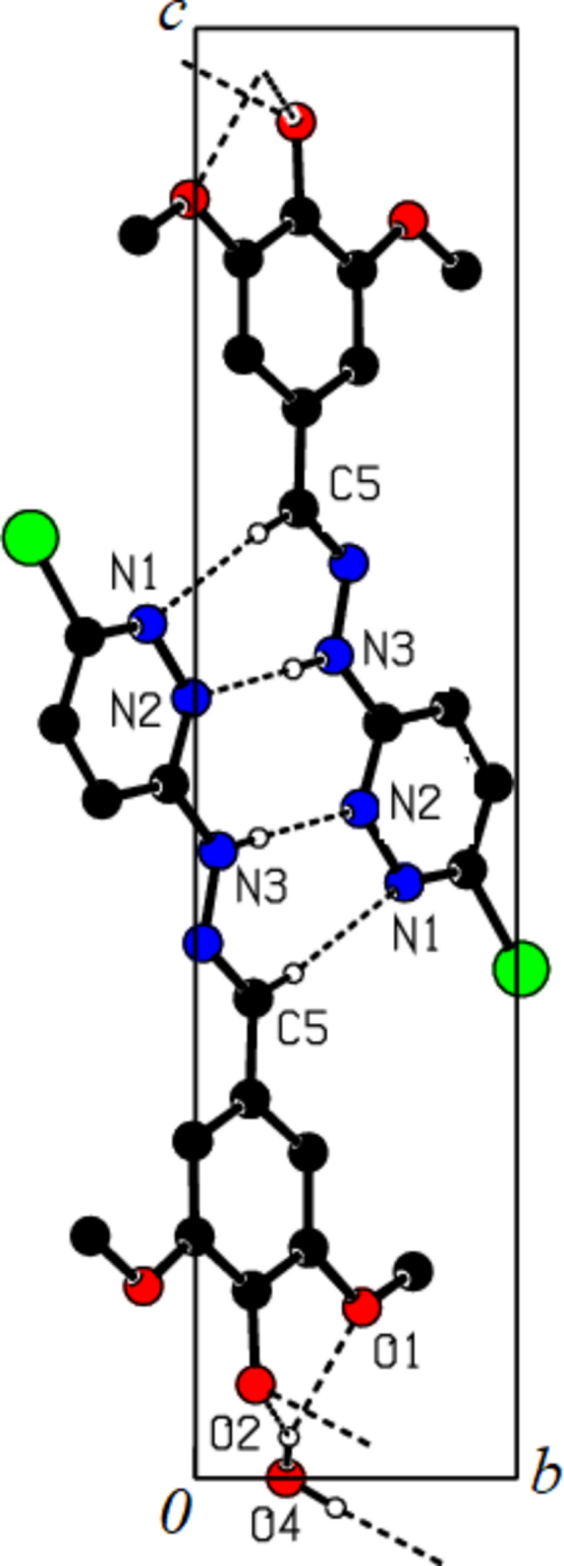
A view along the *a* axis of the crystal packing of title compound. Hydrogen bonds (Table 2 ▸) are shown as dashed lines, and H atoms not involved in hydrogen bonding were omitted for clarity.

Extensive hydrogen bonding plus some van der Waals contacts are the dominant inter­actions in the crystal packing (Table 2 ▸). The C5—H5⋯N1 hydrogen bond generates an inversion dimer with an 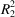 (14) ring motif (Bernstein *et al.*, 1995 ▸); within this larger ring the C3—H3*N*⋯N2 hydrogen bond links the mol­ecules into an 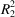
(8) ring motif. The two mol­ecules of one dimer are therefore firmly connected by four hydrogen bonds. In addition, a bifurcated O4—H4*W*⋯(O2, O1)(−*x* + 1, −*y* + 1, −*z* + 2) hydrogen bond is formed between the water hydrogen atom and the hydroxyl and meth­oxy oxygen atoms of an adjacent organic mol­ecule, leading to the formation of an 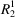
(5) ring. The same water mol­ecule forms another hydrogen bond to the hydroxyl oxygen atom [O4—H5*W*⋯O2(*x*, *y* + 1, *z*)] of a different adjacent mol­ecule. As acceptor, the water forms a hydrogen bond within the asymmetric unit: O2—H2*O*⋯O4. All in all, the water mol­ecules link the hydrogen-bonded dimers throughout the crystal structure into a 3D network, one dimension of which is shown in Fig. 2 ▸. Potentially C—H⋯π inter­actions (Table 2 ▸) and/or off-centre face-to-face π–π stacking inter­action [*Cg*1⋯*Cg*1(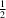 − *x*, −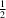 − *y*, 1 − *z*) = 4.374 (2) with slippage of 2.929 Å, where *Cg*1 is the centroid of the N1/N2/C1–C4 ring] may contribute to a two-dimensional substructure. As a result of the presence of a twofold screw axis (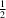 − *x*, −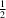 + *y*, 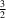 − *z*), which does not lie in the plane of the organic mol­ecule, the latter appears in two orientations relative to the cell dimensions, resulting in a folded sheet arrangement with an inter-planar angle of 88.45°, when all atoms of the two aromatic rings and the hydrazone moiety are used to calculate the planes.

## 4. Database survey

A survey of the Cambridge Structural Database (CSD, Version 5.38; Groom *et al.*, 2016 ▸) for the presence of 3-(2-benzyl­idenehydrazin­yl)pyridazine as the main skeleton revealed three structures as closely related to the title compound. The mol­ecule of DUTQUF (Ather *et al.*, 2010 ▸) is much less planar than the title compound. However, the bidirectional N—H⋯N hydrogen bonding is present here as well, albeit twisted because of the non-planarity. Chlorine acts as a hydrogen-bonding acceptor. In GUTYEB01 (Bakale *et al.*, 2018 ▸), the mol­ecule is also less planar due to the torsion of the nitro group, and the aromatic systems are not as coplanar as in the title compound. It is the only one of the three related compounds that has co-crystallized water. Hydrogen bonding involving water forms dimers of mol­ecules. The three-dimensional hydrogen-bonding network is facilitated by including chloride as hydrogen-bonding acceptor from water O—H, phenyl C—H and hydrazine N—H. Lastly, the KUZSOP (Rafi *et al.*, 2016 ▸) mol­ecule is less planar than the title compound in its general scaffold but more planar than the other two. It is the only one of the three related derivatives that has the exact same hydrogen-bonding pattern for the N—H⋯N contacts as found in the tittle compound. Additional C—H⋯O inter­actions lead to band structures, which protrude indefinitely through the crystal structure and are not connected to any adjacent bands. This means that the hydrogen-bonding network is two-dimensional considering the mol­ecular dimensions and one-dimensional considering the band dimension, which is a unique feature among the four structures. All compounds have N—H⋯N bonds except GUTYEB01, while KUZSOP has overall similar types of hydrogen bonds (C—H⋯N and O—H⋯N) as the title compound. The N—C—N—N torsion angle between the pyridazine ring and hydrazine moiety of the title compound are comparable with those in DUTQUF and GUTYEB01 but differs slightly more from that in KUZSOP. The C—C—N—N torsion angles between the phenyl ring and hydrazine moiety of the title compound are comparable with all three compounds. Most of the bond angles of the title compound have close similarities with all three compounds, except for N—N—H and N—C—N, which may be attributed to the formation of hydrogen bonding involving these atoms.

## 5. Mol­ecular docking studies

The main factors in determining the binding affinity and the efficacy of a new drug mol­ecule are hydrogen bonding and hydro­phobic inter­actions. Hydrogen bonds play an important role in drug-receptor inter­actions, which improve many biological functions (Chen *et al.*, 2016 ▸), while the hydro­phobic inter­actions affect a wide range of biological processes. Hence, mol­ecular docking studies were utilized to predict the *in silico* mol­ecular inter­actions between the compound and the targets, namely, EGFR and HER2 protein receptors. The binding energies of the title compound with these receptors are summarized in Table S2 in the Supporting Information.

**Figure 3:**
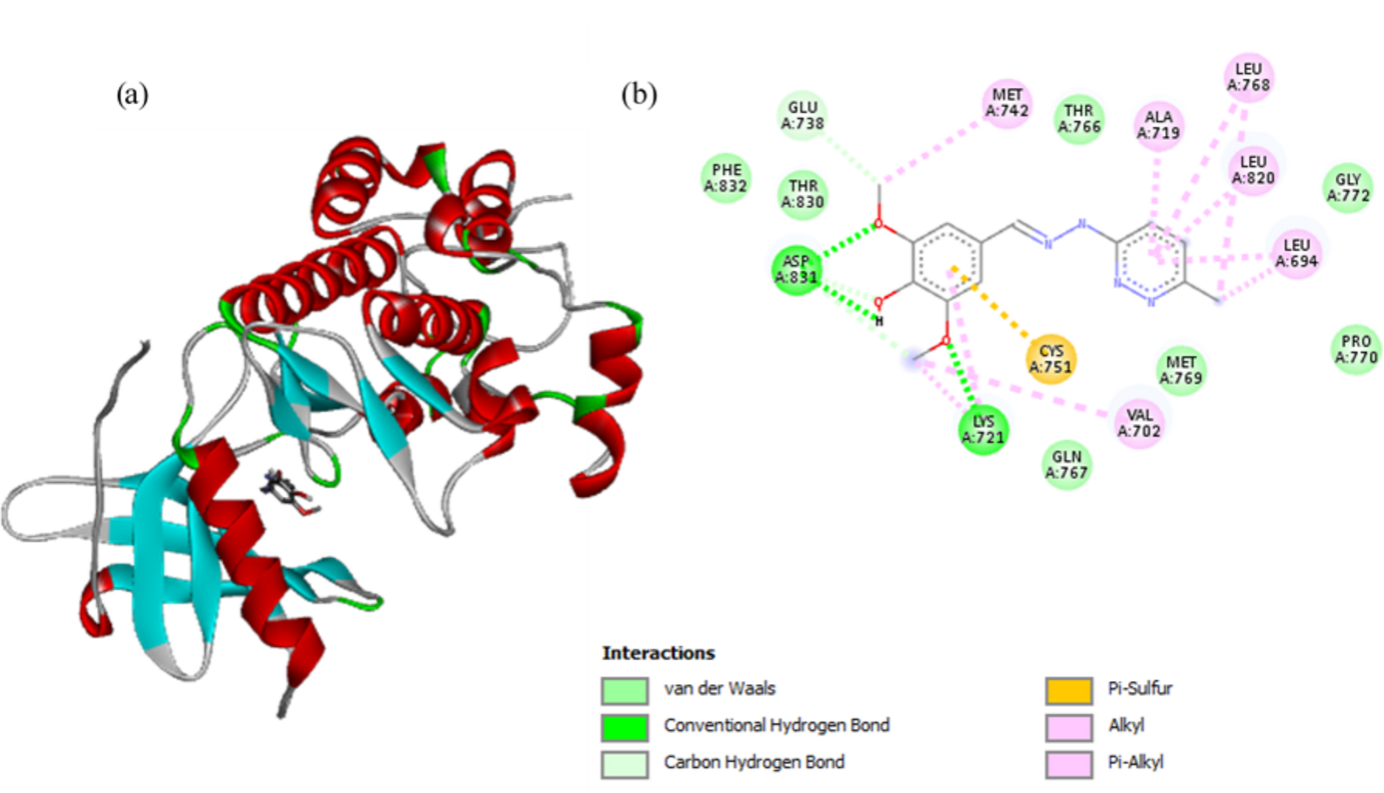
(*a*) Three-dimensional visualization of the binding pose of the compound within the EGFR kinase receptor. (*b*) Two-dimensional ligand–protein inter­action plot of the title compound with the EGFR kinase receptor. Figure prepared using *Discovery Studio Visualizer* (v2021; BIOVIA, 2021 ▸).

4-Hy­droxy-3,5-di­meth­oxy­benzaldehyde (6-chloro­pyrid­azin-3-yl)hydrazone exhibits hydro­phobic inter­actions and hydrogen bonding with both the EGFR and HER2 kinases (Figs. 3 ▸ and 4 ▸). The compound inter­acts with the EGFR receptor through two hydrogen-bonding inter­actions, one between the oxygen atoms of the meth­oxy group with Lys721, and the second between the oxygen atoms of the hydroxyl and meth­oxy groups with Asp831. The compound shows π–sulfur inter­actions with residue Cys751, alkyl inter­actions with residues Leu694, Val702, Lys721 and Met742, as well as van der Waals inter­actions with residues Glu738, Gln767, Met769, Pro770, Gyl772, The830 and Phe832. The compound inter­acts with the HER2 receptor through two hydrogen-bonding inter­actions, the first being the one between the nitro­gen atom of the pyridazine ring with Lys753, and the second between the oxygen atom in the hydroxyl group and Met801. Additionally, the compound has π–sigma inter­actions with residues Ala751, Thr798 and Leu852, alkyl inter­actions with residues Leu785, Leu796, Leu800, Cyc805 and Phe1004, and van der Waals inter­actions with residues Leu726, Val734, Val797 and Thr862.

Though the title compound inter­acts through hydrogen bonds and hydro­phobic inter­actions with both the receptors, it has different binding energy values. The compound has a higher binding energy for the EGFR receptor (−8.43 kJ mol^−1^) than the HER2 receptor (−6.88 kJ mol^−1^). Hence, the compound is tightly bound in the EGFR binding pocket, indicating that it can in principle act as a potent EGFR inhibitor.

## 6. Synthesis and crystallization

An ethano­lic solution (25 mL) of syringaldehyde (0.3642g, 1 mmol) was slowly added to an ethano­lic solution (25 mL) of 3-chloro-6-hydrazinopyridazine (0.289 g, 1 mmol) with constant stirring for 1 h and refluxed for 2 h. The obtained clear solution was allowed to stand and the compound crystallized under slow evaporation. Single crystals of the compound suitable for X-ray analysis were recrystallized by slow evaporation from ethanol/water at room temperature·Yellow solid. Yield: 0.4631 g (70.89%), m.p. 528 K. Selected IR data (KBr, cm^−1^): 3452 ν (OH), 3381 ν (NH), 1695 ν (C=N), 1502 ν (N=N), 1314 ν (Ar—O).

## 7. Refinement

Crystal data, data collection and structure refinement details, are summarized in Table 3 ▸. The water, hydroxyl and NH hydrogen atoms were located in difference-Fourier maps and freely refined. The C-bound H atoms were positioned geometrically and constrained to ride on their parent atoms, with C—H =0.95–0.98 Â, and with *U*_iso_ = 1.5*U*_eq_(C) for methyl H atoms and 1.2*U*_eq_(C) for other H atoms.

## Supplementary Material

Crystal structure: contains datablock(s) global, I. DOI: 10.1107/S205698902500252X/yz2065sup1.cif

Structure factors: contains datablock(s) I. DOI: 10.1107/S205698902500252X/yz2065Isup2.hkl

The supporting information contains the experimental section, molecular docking procedure, IR spectral characterization, additional tables and figure. DOI: 10.1107/S205698902500252X/yz2065sup3.docx

Supporting information file. DOI: 10.1107/S205698902500252X/yz2065Isup4.cml

CCDC reference: 2342583

Additional supporting information:  crystallographic information; 3D view; checkCIF report

## Figures and Tables

**Figure 4:**
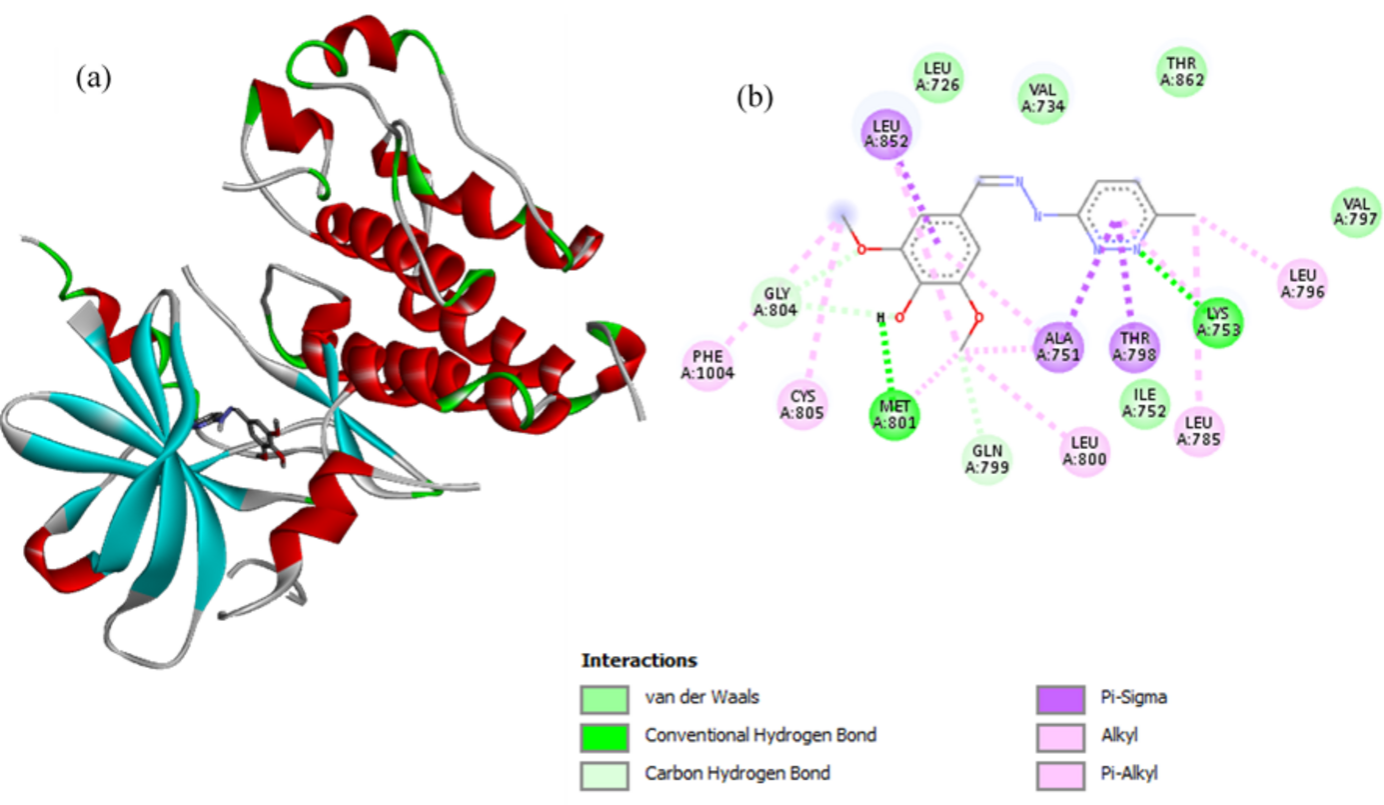
(*a*) Three-dimensional visualization of the binding pose of the compound within the HER2 kinase receptor. (*b*) Two-dimensional ligand–protein inter­action plot of the title compound with the HER2 kinase receptor. Figure prepared using *Discovery Studio Visualizer* (v2021; BIOVIA, 2021 ▸).

**Table 1:** Selected geometric parameters (Å, °)

C4—N3	1.353 (2)	N1—N2	1.350 (2)
C5—N4	1.278 (2)	N3—N4	1.3769 (17)
			
C4—N3—N4	121.38 (15)	C5—N4—N3	113.84 (14)
			
N4—C5—C6—C11	1.3 (2)	C4—N3—N4—C5	−178.52 (15)
C3—C4—N3—N4	−2.2 (3)	C7—C8—O1—C13	8.3 (3)
C6—C5—N4—N3	−178.31 (14)	C11—C10—O3—C12	5.9 (3)

**Table 2:** Hydrogen-bond geometry (Å, °)

*D*—H⋯*A*	*D*—H	H⋯*A*	*D*⋯*A*	*D*—H⋯*A*
C5—H5⋯N1^i^	0.93	2.60	3.519 (2)	172
C12—H12*A*⋯N4^ii^	0.96	2.66	3.369 (2)	132
O2—H2*O*⋯O4	0.85 (2)	1.84 (2)	2.668 (2)	164 (2)
N3—H3*N*⋯N2^i^	0.87 (2)	2.12 (2)	2.993 (2)	178.3 (18)
O4—H4*W*⋯O1^iii^	0.79 (3)	2.23 (3)	2.9254 (19)	146 (3)
O4—H4*W*⋯O2^iii^	0.79 (3)	2.55 (3)	3.239 (2)	147 (3)
O4—H5*W*⋯O2^iv^	0.85 (3)	2.08 (3)	2.914 (2)	169 (3)
C12—H12*B*⋯*Cg*(C6–C11)^v^	0.96	2.87	3.671 (1)	142
C13—H13*B*⋯*Cg*(C6–C11)^iv^	0.96	2.90	3.666 (1)	138

Symmetry codes: (i) 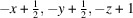; (ii) 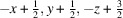; (iii) 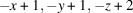; (iv) 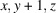; (v) 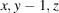.

**Table 3:** Experimental details

Crystal data	
Chemical formula	C_13_H_13_ClN_4_O_3_·H_2_O
*M*_r_	326.74
Crystal system, space group	Monoclinic, *C*2/*c*
Temperature (K)	298
*a*, *b*, *c* (Å)	32.1977 (19), 4.6431 (3), 26.400 (2)
β (°)	127.495 (2)
*V* (Å^3^)	3131.4 (4)
*Z*	8
Radiation type	Mo *K*α
μ (mm^−1^)	0.27
Crystal size (mm)	0.45 × 0.25 × 0.10

Data collection	
Diffractometer	Bruker D8 VENTURE with PHOTON II detector
Absorption correction	Multi-scan (*SADABS*; Krause *et al.*, 2015 ▸)
*T*_min_, *T*_max_	0.580, 0.746
No. of measured, independent and observed [*I* > 2σ(*I*)] reflections	32222, 3867, 3259
*R*_int_	0.052
(sin θ/λ)_max_ (Å^−1^)	0.666

Refinement	
*R*[*F*^2^ > 2σ(*F*^2^)], *wR*(*F*^2^), *S*	0.049, 0.132, 1.07
No. of reflections	3867
No. of parameters	215
H-atom treatment	H atoms treated by a mixture of independent and constrained refinement
Δρ_max_, Δρ_min_ (e Å^−3^)	0.32, −0.27

Computer programs: *APEX3*, *SAINT* and *XPREP* (Bruker, 2016 ▸), *SHELXT2018/2* (Sheldrick, 2015*a* ▸), *SHELXL2019/3* (Sheldrick, 2015*b* ▸), *ORTEP-3 for Windows* and *WinGX* (Farrugia, 2012 ▸), *PLATON* (Spek, 2009 ▸) and *publCIF* (Westrip, 2010 ▸).

## supplementary crystallographic information

### 4-Hydroxy-3,5-dimethoxybenzaldehyde (6-chloropyridazin-3-yl)hydrazone monohydrate Crystal data

**Table:**

C_13_H_13_ClN_4_O_3_·H_2_O	*F*(000) = 1360
*M**_r_* = 326.74	*D*_x_ = 1.386 Mg m^−^^3^
Monoclinic, *C*2/*c*	Mo *K*α radiation, λ = 0.71073 Å
*a* = 32.1977 (19) Å	Cell parameters from 9970 reflections
*b* = 4.6431 (3) Å	θ = 2.5–28.2°
*c* = 26.400 (2) Å	µ = 0.27 mm^−^^1^
β = 127.495 (2)°	*T* = 298 K
*V* = 3131.4 (4) Å^3^	Block, brown
*Z* = 8	0.45 × 0.25 × 0.10 mm

### 4-Hydroxy-3,5-dimethoxybenzaldehyde (6-chloropyridazin-3-yl)hydrazone monohydrate Data collection

**Table:**

Bruker D8 VENTURE diffractometer equipped with PHOTON II detector	3259 reflections with *I* > 2σ(*I*)
Radiation source: fine-focus sealed tube	*R*_int_ = 0.052
ω and φ scan	θ_max_ = 28.3°, θ_min_ = 3.2°
Absorption correction: multi-scan (SADABS; Krause *et al.*, 2015)	*h* = −42→42
*T*_min_ = 0.580, *T*_max_ = 0.746	*k* = −6→4
32222 measured reflections	*l* = −35→35
3867 independent reflections	

### 4-Hydroxy-3,5-dimethoxybenzaldehyde (6-chloropyridazin-3-yl)hydrazone monohydrate Refinement

**Table:**

Refinement on *F*^2^	Primary atom site location: dual
Least-squares matrix: full	Hydrogen site location: mixed
*R*[*F*^2^ > 2σ(*F*^2^)] = 0.049	H atoms treated by a mixture of independent and constrained refinement
*wR*(*F*^2^) = 0.132	*w* = 1/[σ^2^(*F*_o_^2^) + (0.0564*P*)^2^ + 2.0701*P*] where *P* = (*F*_o_^2^ + 2*F*_c_^2^)/3
*S* = 1.07	(Δ/σ)_max_ = 0.001
3867 reflections	Δρ_max_ = 0.32 e Å^−^^3^
215 parameters	Δρ_min_ = −0.27 e Å^−^^3^
0 restraints	

### 4-Hydroxy-3,5-dimethoxybenzaldehyde (6-chloropyridazin-3-yl)hydrazone monohydrate Special details

**Table:**

Geometry. All esds (except the esd in the dihedral angle between two l.s. planes) are estimated using the full covariance matrix. The cell esds are taken into account individually in the estimation of esds in distances, angles and torsion angles; correlations between esds in cell parameters are only used when they are defined by crystal symmetry. An approximate (isotropic) treatment of cell esds is used for estimating esds involving l.s. planes.

### 4-Hydroxy-3,5-dimethoxybenzaldehyde (6-chloropyridazin-3-yl)hydrazone monohydrate Fractional atomic coordinates and isotropic or equivalent isotropic displacement parameters (Å^2^)

**Table:**

	*x*	*y*	*z*	*U*_iso_*/*U*_eq_	
C1	0.12978 (7)	0.8456 (4)	0.41979 (9)	0.0544 (4)	
C2	0.14422 (8)	0.9256 (4)	0.47956 (10)	0.0612 (5)	
H2	0.125598	1.064179	0.483681	0.073*	
C3	0.18687 (8)	0.7917 (4)	0.53169 (9)	0.0570 (4)	
H3	0.198656	0.837253	0.572787	0.068*	
C4	0.21223 (6)	0.5831 (4)	0.52099 (7)	0.0452 (4)	
C5	0.31793 (6)	0.3204 (4)	0.66896 (7)	0.0473 (4)	
H5	0.328952	0.194971	0.651841	0.057*	
C6	0.34738 (6)	0.3305 (4)	0.73833 (7)	0.0424 (3)	
C7	0.39076 (6)	0.1498 (4)	0.77521 (7)	0.0469 (4)	
H7	0.400366	0.030334	0.755628	0.056*	
C8	0.41951 (6)	0.1486 (4)	0.84109 (7)	0.0448 (3)	
C9	0.40516 (5)	0.3252 (3)	0.87054 (6)	0.0411 (3)	
C10	0.36127 (6)	0.5033 (3)	0.83331 (7)	0.0397 (3)	
C11	0.33270 (6)	0.5085 (3)	0.76741 (7)	0.0411 (3)	
H11	0.303949	0.630052	0.742856	0.049*	
C12	0.30407 (7)	0.8281 (5)	0.83303 (9)	0.0615 (5)	
H12A	0.301109	0.929493	0.862401	0.092*	
H12B	0.305110	0.964118	0.806431	0.092*	
H12C	0.274442	0.703171	0.806886	0.092*	
C13	0.48434 (8)	−0.1772 (5)	0.85738 (12)	0.0732 (6)	
H13A	0.514242	−0.283563	0.891218	0.110*	
H13B	0.458548	−0.308440	0.825325	0.110*	
H13C	0.494847	−0.047217	0.838793	0.110*	
N1	0.15369 (6)	0.6503 (4)	0.41076 (7)	0.0542 (4)	
N2	0.19538 (6)	0.5134 (3)	0.46188 (6)	0.0509 (3)	
N3	0.25458 (6)	0.4304 (4)	0.56743 (6)	0.0515 (4)	
N4	0.27767 (5)	0.4770 (3)	0.63098 (6)	0.0470 (3)	
O1	0.46277 (5)	−0.0183 (3)	0.88222 (6)	0.0642 (4)	
O2	0.43409 (5)	0.3129 (3)	0.93550 (5)	0.0548 (3)	
O3	0.35056 (4)	0.6626 (3)	0.86749 (5)	0.0550 (3)	
O4	0.44334 (6)	0.7811 (3)	1.00029 (6)	0.0619 (4)	
Cl1	0.07736 (2)	1.01427 (14)	0.35124 (3)	0.0811 (2)	
H2O	0.4312 (9)	0.472 (5)	0.9495 (11)	0.072 (7)*	
H3N	0.2689 (8)	0.304 (4)	0.5582 (9)	0.054 (5)*	
H4W	0.4738 (12)	0.791 (6)	1.0283 (15)	0.100 (10)*	
H5W	0.4362 (10)	0.937 (6)	0.9796 (13)	0.084 (8)*	

### 4-Hydroxy-3,5-dimethoxybenzaldehyde (6-chloropyridazin-3-yl)hydrazone monohydrate Atomic displacement parameters (Å^2^)

**Table:**

	*U*^11^	*U*^22^	*U*^33^	*U*^12^	*U*^13^	*U*^23^
C1	0.0465 (8)	0.0610 (10)	0.0545 (9)	−0.0077 (8)	0.0300 (8)	0.0089 (8)
C2	0.0656 (11)	0.0575 (10)	0.0721 (12)	−0.0019 (9)	0.0478 (10)	0.0019 (9)
C3	0.0697 (11)	0.0578 (10)	0.0532 (9)	−0.0107 (9)	0.0425 (9)	−0.0058 (8)
C4	0.0478 (8)	0.0523 (9)	0.0386 (7)	−0.0127 (7)	0.0279 (7)	−0.0030 (6)
C5	0.0485 (8)	0.0610 (10)	0.0361 (7)	−0.0106 (7)	0.0277 (7)	−0.0088 (7)
C6	0.0409 (7)	0.0515 (9)	0.0347 (7)	−0.0104 (6)	0.0230 (6)	−0.0068 (6)
C7	0.0444 (8)	0.0545 (9)	0.0436 (8)	−0.0056 (7)	0.0278 (7)	−0.0133 (7)
C8	0.0360 (7)	0.0463 (8)	0.0431 (8)	0.0007 (6)	0.0194 (6)	−0.0060 (6)
C9	0.0371 (7)	0.0455 (8)	0.0311 (7)	−0.0007 (6)	0.0158 (6)	−0.0040 (6)
C10	0.0356 (7)	0.0461 (8)	0.0331 (7)	−0.0010 (6)	0.0187 (6)	−0.0054 (6)
C11	0.0350 (7)	0.0492 (8)	0.0320 (7)	−0.0006 (6)	0.0168 (6)	−0.0008 (6)
C12	0.0467 (9)	0.0736 (13)	0.0581 (10)	0.0132 (9)	0.0288 (8)	−0.0081 (9)
C13	0.0618 (11)	0.0681 (13)	0.0876 (15)	0.0153 (10)	0.0444 (11)	−0.0102 (11)
N1	0.0475 (7)	0.0687 (10)	0.0404 (7)	−0.0070 (7)	0.0237 (6)	0.0036 (7)
N2	0.0504 (7)	0.0645 (9)	0.0357 (6)	−0.0045 (6)	0.0251 (6)	−0.0007 (6)
N3	0.0536 (8)	0.0668 (9)	0.0330 (6)	−0.0029 (7)	0.0257 (6)	−0.0052 (6)
N4	0.0499 (7)	0.0603 (8)	0.0323 (6)	−0.0128 (6)	0.0258 (6)	−0.0063 (6)
O1	0.0499 (7)	0.0685 (8)	0.0518 (7)	0.0191 (6)	0.0193 (6)	−0.0089 (6)
O2	0.0540 (7)	0.0569 (7)	0.0317 (5)	0.0128 (6)	0.0148 (5)	−0.0027 (5)
O3	0.0473 (6)	0.0731 (8)	0.0345 (5)	0.0169 (6)	0.0197 (5)	−0.0053 (5)
O4	0.0582 (8)	0.0642 (9)	0.0379 (6)	−0.0048 (7)	0.0161 (6)	−0.0042 (6)
Cl1	0.0572 (3)	0.0952 (4)	0.0770 (4)	0.0074 (3)	0.0337 (3)	0.0300 (3)

### 4-Hydroxy-3,5-dimethoxybenzaldehyde (6-chloropyridazin-3-yl)hydrazone monohydrate Geometric parameters (Å, º)

**Table:**

C1—N1	1.302 (2)	C9—C10	1.399 (2)
C1—C2	1.397 (3)	C10—O3	1.3636 (18)
C1—Cl1	1.7374 (19)	C10—C11	1.3874 (19)
C2—C3	1.366 (3)	C11—H11	0.9300
C2—H2	0.9300	C12—O3	1.415 (2)
C3—C4	1.402 (3)	C12—H12A	0.9600
C3—H3	0.9300	C12—H12B	0.9600
C4—N2	1.343 (2)	C12—H12C	0.9600
C4—N3	1.353 (2)	C13—O1	1.418 (2)
C5—N4	1.278 (2)	C13—H13A	0.9600
C5—C6	1.463 (2)	C13—H13B	0.9600
C5—H5	0.9300	C13—H13C	0.9600
C6—C11	1.392 (2)	N1—N2	1.350 (2)
C6—C7	1.396 (2)	N3—N4	1.3769 (17)
C7—C8	1.386 (2)	N3—H3N	0.87 (2)
C7—H7	0.9300	O2—H2O	0.85 (2)
C8—O1	1.3706 (19)	O4—H4W	0.79 (3)
C8—C9	1.388 (2)	O4—H5W	0.85 (3)
C9—O2	1.3664 (17)		
			
N1—C1—C2	124.38 (17)	O3—C10—C9	114.20 (12)
N1—C1—Cl1	115.54 (14)	C11—C10—C9	120.45 (13)
C2—C1—Cl1	120.07 (16)	C10—C11—C6	119.51 (14)
C3—C2—C1	117.16 (18)	C10—C11—H11	120.2
C3—C2—H2	121.4	C6—C11—H11	120.2
C1—C2—H2	121.4	O3—C12—H12A	109.5
C2—C3—C4	117.59 (17)	O3—C12—H12B	109.5
C2—C3—H3	121.2	H12A—C12—H12B	109.5
C4—C3—H3	121.2	O3—C12—H12C	109.5
N2—C4—N3	113.48 (16)	H12A—C12—H12C	109.5
N2—C4—C3	121.83 (16)	H12B—C12—H12C	109.5
N3—C4—C3	124.68 (15)	O1—C13—H13A	109.5
N4—C5—C6	122.62 (15)	O1—C13—H13B	109.5
N4—C5—H5	118.7	H13A—C13—H13B	109.5
C6—C5—H5	118.7	O1—C13—H13C	109.5
C11—C6—C7	120.26 (13)	H13A—C13—H13C	109.5
C11—C6—C5	122.03 (15)	H13B—C13—H13C	109.5
C7—C6—C5	117.69 (14)	C1—N1—N2	118.93 (15)
C8—C7—C6	119.86 (14)	C4—N2—N1	120.08 (15)
C8—C7—H7	120.1	C4—N3—N4	121.38 (15)
C6—C7—H7	120.1	C4—N3—H3N	121.0 (13)
O1—C8—C7	125.21 (14)	N4—N3—H3N	117.6 (13)
O1—C8—C9	114.48 (13)	C5—N4—N3	113.84 (14)
C7—C8—C9	120.32 (14)	C8—O1—C13	118.39 (15)
O2—C9—C8	118.24 (13)	C9—O2—H2O	109.7 (16)
O2—C9—C10	122.15 (13)	C10—O3—C12	117.64 (12)
C8—C9—C10	119.59 (13)	H4W—O4—H5W	104 (3)
O3—C10—C11	125.35 (13)		
			
N1—C1—C2—C3	1.1 (3)	C8—C9—C10—C11	−1.3 (2)
Cl1—C1—C2—C3	−177.81 (14)	O3—C10—C11—C6	−178.86 (15)
C1—C2—C3—C4	−0.7 (3)	C9—C10—C11—C6	1.3 (2)
C2—C3—C4—N2	−0.6 (3)	C7—C6—C11—C10	−0.5 (2)
C2—C3—C4—N3	−179.46 (17)	C5—C6—C11—C10	178.22 (14)
N4—C5—C6—C11	1.3 (2)	C2—C1—N1—N2	−0.1 (3)
N4—C5—C6—C7	−179.98 (15)	Cl1—C1—N1—N2	178.86 (12)
C11—C6—C7—C8	−0.3 (2)	N3—C4—N2—N1	−179.38 (14)
C5—C6—C7—C8	−179.12 (15)	C3—C4—N2—N1	1.6 (2)
C6—C7—C8—O1	−179.85 (15)	C1—N1—N2—C4	−1.3 (2)
C6—C7—C8—C9	0.4 (2)	N2—C4—N3—N4	178.88 (14)
O1—C8—C9—O2	−1.0 (2)	C3—C4—N3—N4	−2.2 (3)
C7—C8—C9—O2	178.82 (15)	C6—C5—N4—N3	−178.31 (14)
O1—C8—C9—C10	−179.37 (15)	C4—N3—N4—C5	−178.52 (15)
C7—C8—C9—C10	0.4 (2)	C7—C8—O1—C13	8.3 (3)
O2—C9—C10—O3	0.5 (2)	C9—C8—O1—C13	−171.94 (17)
C8—C9—C10—O3	178.88 (15)	C11—C10—O3—C12	5.9 (3)
O2—C9—C10—C11	−179.61 (14)	C9—C10—O3—C12	−174.30 (16)

### 4-Hydroxy-3,5-dimethoxybenzaldehyde (6-chloropyridazin-3-yl)hydrazone monohydrate Hydrogen-bond geometry (Å, º)

**Table:**

*D*—H···*A*	*D*—H	H···*A*	*D*···*A*	*D*—H···*A*
C5—H5···N1^i^	0.93	2.60	3.519 (2)	172
C12—H12*A*···N4^ii^	0.96	2.66	3.369 (2)	132
O2—H2*O*···O4	0.85 (2)	1.84 (2)	2.668 (2)	164 (2)
N3—H3*N*···N2^i^	0.87 (2)	2.12 (2)	2.993 (2)	178.3 (18)
O4—H4*W*···O1^iii^	0.79 (3)	2.23 (3)	2.9254 (19)	146 (3)
O4—H4*W*···O2^iii^	0.79 (3)	2.55 (3)	3.239 (2)	147 (3)
O4—H5*W*···O2^iv^	0.85 (3)	2.08 (3)	2.914 (2)	169 (3)
C12—H12*B*···*Cg*(C6–C11)^v^	0.96	2.87	3.671 (1)	142
C13—H13*B*···*Cg*(C6–C11)^iv^	0.96	2.90	3.666 (1)	138

Symmetry codes: (i) −*x*+1/2, −*y*+1/2, −*z*+1; (ii) −*x*+1/2, *y*+1/2, −*z*+3/2; (iii) −*x*+1, −*y*+1, −*z*+2; (iv) *x*, *y*+1, *z*; (v) *x*, *y*−1, *z*.
