# Supplementary material for: Synthesis, crystal structure, and in silico mol­ecular docking studies of 4-hy­droxy-3,5-di­meth­oxy­benzaldehyde (6-chloro­pyridazin-3-yl)hydrazone monohydrate
Source: Acta Crystallogr E Crystallogr Commun. 2025 Mar 25;81(Pt 4):336–40. doi: 10.1107/S205698902500252X (PMC11974334; doi:10.1107/S205698902500252X)
Supplement: Supplementary file 3 [file e-81-00336-sup3.docx]

Supporting information

**Synthesis, Crystal structure, and *in silico* Molecular docking studies of 4-hydroxy-3,5-dimethoxybenzaldehyde (6-chloropyridazin-3-yl)hydrazone hydrate**

**Ummer Muhammed Rafi,^a*^ NizamMohideen,^b^ Noorulla Mohammed Nazrudeen,^a^ Moolan Khaja Abubacker Sidhik.^a^**

*^a^Post-Graduate and Research Department of Chemistry, The New College, University of Madras, Chennai 600 014, India. ^b^ Department of Physics, The New College, University of Madras, Chennai 600 014, India.*

*Corresponding author, email: [muhammedrafi@thenewcollege.edu.in](mailto:muhammedrafi@thenewcollege.edu.in).

**Experimental Section**

3-Chloro-6-hydrazinopyridazine and syringaldehyde were purchased from Aldrich. The solvents, which are used for the compound synthesis were dried and purified using the standard procedure (Armarego & Perrin, 1996). Electrothermal capillary apparatus was used to determine the melting point. Perkin-Elmer 297 spectrophotometer was used to record IR spectra. The compound (**1**) was synthesized by following the procedure as described in the literature (Rafi *et al.*, 2018).

**Molecular docking studies**

The molecular docking studies were done using AutoDock version 4.2.5.1 (Morris *et al.*, 2009) and AutoDock Tools version 1.5.6 (Sanner, 1999) docking programs. The crystal structure of EGFR (PDB ID: 1M17) and HER2 (PDB ID: 3RCD) was downloaded from the Research Collaboratory for Structural Bioinformatics (RCSB) protein data bank (PDB). The docking was performed employing the procedure as reported previously (Rafi *et al.*, 2016). The grid box size was 40 × 40 × 40 along with the X, Y, and Z axes for both enzymes, and the coordinates of the central grid point of the map have been set to be 18.74, 31.83, and 11.62 for EGFR and 12.48, 2.96, and 28.01 for HER2. The lowest energy docked conformation was selected, and BIOVIA Discovery Studio Visualizer v2021 (BIOVIA, 2021) was used to visualize the intermolecular interactions between the receptors and compound in 2D and 3D representations.

**IR Spectroscopy**

The formation of the compound was characterized by IR spectrum. The IR spectrum for the compound is shown in Fig. S1 and their characteristic peak assignments are given in Table S1. The ν(C=N) stretching vibration of azomethine group at 1695cm^-1^, ν(Ar-O) stretching vibration of phenolic -OH group at 1314cm^-1^ and ν(N=N) stretching vibration of the unsymmetrical pyridazine ring at 1502cm^-1^ has been observed for the ligand.

**References**

Armarego, W. L. F. & Perrin, D. D. (1996). '*Purification of Laboratory Chemicals*', Pergamon press. Fourth ed, Oxford.

BIOVIA, Discovery Studio Modeling Environment, Release 2021, San Diego: Dassault Systèmes, (2021).

Morris, G. M., Huey, R., Lindstrom, W., Sanner, M. F., Belew, R. K., Goodsell, D. S. & Olson, A. J. (2009). *J. Comput. Chem.* **16** 2785–2791.

Rafi, U. M., Mahendiran, D., Devi, V. G., Doble, M. & Rahiman, A. K. (2018). *Inorg. Chim. Acta* **482** 160–169.

Rafi, U. M., Mahendiran, D., Haleel, A. K., Nankar, R. P., Doble, M. & Rahiman, A. K. (2016). *New J. Chem.* **40** 2451–2465.

Sanner, M. F. (1999). *J. Mol. Graph Model* **17** 57–61.

**Table S1** Selected IR data of the title compound.



| Compound | ν(OH) | ν(C=N) | ν(Ar−O) | ν( N=N) |
| --- | --- | --- | --- | --- |
|  | 3485 | 1695 | 1314 | 1502 |

**Table S2**. Determination of binding energy of the title compound with the EGFR and HER2 proteins using docking studies.

| Receptor | Final intermolecular energy  kcal/mol | | | Final total internal energy (2) kcal/mol | Torsional free energy  (3) kcal/mol | Unbound system's energy (4) kcal/mol | Estimated free energy of binding  [(1)+(2)+(3)–(4)] kcal/mol |
| --- | --- | --- | --- | --- | --- | --- | --- |
|  | vdW + H bond + dissolving energy | Electrostatic Energy | Total  (1) |  |  |  |  |
| EGFR | –9.67 | –0.55 | –10.22 | –1.21 | +1.79 | –1.21 | –8.43 |
| HER2 | –8.57 | –0.10 | –8.67 | –1.27 | +1.79 | –1.27 | –6.88 |


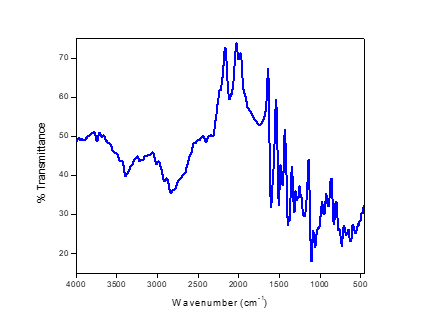


**Figure S1** IR spectrum of the title compound.
